# The Association Between Heatmap Position and the Diagnostic Accuracy of Artificial Intelligence for Colorectal Polyp Diagnosis

**DOI:** 10.3390/cancers17101620

**Published:** 2025-05-10

**Authors:** Ayla Thijssen, Nikoo Dehghani, Ruud W. M. Schrauwen, Eric T. P. Keulen, Eveline J. A. Rondagh, Mark H. P. van Avesaat, Khalida Soufidi, Ankie Reumkens, Paul H. A. Bours, Quirine E. W. van der Zander, Peter H. N. de With, Bjorn Winkens, Fons van der Sommen, Erik J. Schoon

**Affiliations:** 1 Department of Gastroenterology and Hepatology, Maastricht University Medical Center+, 6202 AZ Maastricht, The Netherlands; 2 GROW Research Institute for Oncology and Reproduction, Maastricht University, 6202 AZ Maastricht, The Netherlands; 3 Department of Electrical Engineering, Eindhoven University of Technology, 5600 MB Eindhoven, The Netherlands; 4 Department of Gastroenterology and Hepatology, Bernhoven Hospital, Nistelrodeseweg 10, 5406 PT Uden, The Netherlands; 5 Department of Gastroenterology and Hepatology, Zuyderland Medical Center, Dr. H. van der Hoffplein 1, 6162 AP Sittard-Geleen, The Netherlands; 6 Department of Methodology and Statistics, Maastricht University, 6202 AZ Maastricht, The Netherlands; 7 CAPHRI, Care and Public Health Research Institute, Maastricht University, 6202 AZ Maastricht, The Netherlands; 8 Department of Gastroenterology and Hepatology, Catharina Hospital, Michelangelolaan 2, 5623 EJ Eindhoven, The Netherlands

**Keywords:** colorectal polyps, colonoscopy, computer-aided diagnosis, visually explainable artificial intelligence

## Abstract

Background/Objectives: Artificial intelligence (AI) algorithms for diagnosing colorectal polyps are emerging but not yet widely used. Trust in AI is lacking and could be improved by visually explainable AI, such as heatmaps. This study aims to investigate the association between heatmap position and AI accuracy for the endoscopic characterization of colorectal polyps. Methods: Four AI algorithms diagnosed 2133 prospectively collected images of 376 colorectal polyps from two hospitals, using histopathology as the gold standard. Heatmap position was compared to the human-annotated polyp position. Generalized estimating equations were used to assess the association between heatmap position and a correct AI diagnosis. Results: Higher percentages of heatmap covering the colorectal polyp were associated with correct diagnoses in all four algorithms (OR 1.013 [95% CI 1.006–1.019], OR 1.025 [95% CI 1.011–1.039], OR 1.038 [95% CI 1.024–1.053], and OR 1.039 [95% CI 1.020–1.058]—all *p* < 0.001). A higher percentage of polyp not covered by heatmap was associated with a correct diagnosis of Algorithm 1 (OR 1.006 [95% CI 1.003–1.010], *p* < 0.001), while in Algorithm 2, a lower percentage was associated with a correct diagnosis (OR 0.992 [95% CI 0.985–1.000], *p* 0.044). Algorithms 3 and 4 showed negative, but not statistically significant, associations. Conclusions: Higher percentages of heatmap covering the polyp were associated with correct diagnoses of four AI algorithms. This indicates that it is clinically relevant to strive for AI predictions with heatmaps covering as much colorectal polyp tissue as possible. Knowing how to interpret heatmaps could increase trust in AI and, with that, benefit the implementation of AI in clinical practice.

## Simple Summary

Artificial intelligence (AI) shows great potential to improve the diagnosis of colorectal polyps, precursors of colorectal cancer, during endoscopy. However, AI is not widely used for this purpose yet. Among other things, this is caused by a lack of trust in AI. Explainable AI could increase trust in AI by creating more transparent outcomes. Heatmaps are an example of visually explainable AI. Heatmaps highlight the target area of an image used by the AI algorithm to make a diagnosis. This study aimed to investigate the association between heatmap position and AI accuracy for the diagnosis of colorectal polyps on endoscopic images. The higher the percentage of heatmap covering the colorectal polyp, the better the AI accuracy was in four different AI algorithms. With this knowledge, doctors using AI in colonoscopy know that it is relevant to strive for an AI diagnosis with a heatmap covering as much colorectal polyp tissue as possible.

## 1. Introduction

The application of artificial intelligence (AI) to improve the endoscopic optical diagnosis of colorectal polyps, precursors of colorectal cancer, is a relevant topic in current gastroenterology research. Computer-aided diagnosis (CADx) systems are achieving promising results in distinguishing benign from premalignant colorectal polyps [1,2]. Improved optical diagnosis using CADx systems could facilitate the application of the ‘diagnose-and-leave’ and ‘resect-and-discard’ treatment strategies in clinical practice [3,4]. With these strategies, diminutive (≤5 mm) hyperplastic polyps could be left in situ, and diminutive adenomas could be resected without histopathological examination, reducing post-polypectomy complications and healthcare costs.

Nevertheless, CADx systems for diminutive colorectal polyps are not being widely used in daily clinical practice yet. Among other things, this is likely a result of a lack of trust in AI by endoscopists. Previous research has shown that trust is a primary mediator for the acceptance of AI in clinical practice by endoscopists [5]. Current CADx systems generate diagnoses using methods that are not transparent. This “black box” characteristic of AI algorithms can negatively influence AI trustworthiness [6].

Explainable AI is a solution to enhance transparency and provide insight into the reasoning behind an AI diagnosis, by explaining the internal decision-making process of neural networks in an easy-to-understand way [7,8]. Discarding an informative CADx prediction due to a lack of trust, called under-reliance, could be limited with explainable AI [9]. Additionally, explainable AI might make the application of CADx systems in clinical practice safer by decreasing over-reliance and could enable the optimal interaction between CADx systems and their users, because endoscopists can make substantiated decisions to either agree with a CADx system diagnosis or not. If the endoscopists are outperforming CADx systems, explainable AI can also identify the CADx system failure better, guiding the future improvement of the CADx system to come closer to implementation in clinical practice [10]. Furthermore, less experienced endoscopists can learn from CADx diagnoses containing explainable AI. Therefore, the explainability of AI algorithms might play a key role in their adoption in clinical practice.

Textual descriptions of colorectal polyp features are a form of explainable AI and have the potential to increase the understanding of AI predictions [11]. Alternatively, heatmaps are visual explanations highlighting the target area of an image used by the AI algorithm to make a prediction [10,12,13]. Saliency mapping can be used to see which information of the image is used by a classification model to make its prediction (e.g., Grad-CAM, Gradient-weighted class activation mapping [10]).

In practice, endoscopic images of colorectal polyps often contain additional information such as the surrounding mucosa or colon lumen. When using a CADx system on these endoscopic images, heatmaps can be placed on the colorectal polyp, possibly additionally on surrounding features, or even only on surrounding features. In order to use heatmaps as a form of explainable AI to increase trust in AI, knowledge regarding the correct interpretation of heatmaps is required. In other words, endoscopists would benefit from evidence to learn if a heatmap needs to cover the colorectal polyp in order to increase the chances of an accurate characterization. This knowledge could guide the clinical use of CADx systems with heatmaps by clarifying whether it would be helpful to reposition the endoscope if the heatmap does not cover the polyp correctly. Although intuitively it would seem most logical that heatmaps need to cover (solely) the colorectal polyp to make the most accurate prediction, this has not yet been proven in an experimental setting. The performance of explanations is rarely tested, especially not by scoring explanations from a human perspective [14].

This study aims to investigate the association between heatmap position and the diagnostic accuracy of four AI algorithms for the characterization of colorectal polyps on endoscopic images.

## 2. Materials and Methods

### 2.1. Data Collection and Preprocessing

In this study, two datasets containing images of colorectal polyps were collected prospectively.

At Bernhoven Hospital Uden, the Netherlands, a dataset with Fujifilm (Tokyo, Japan) videos was collected between April 2022 and January 2024. Videos were taken by one endoscopist. For this study, frames of colorectal lesions were captured from these videos. If possible, nine unique images were extracted for each colorectal polyp—three images in each image enhancement technique (HDWL, high-definition white light; BLI, blue light imaging; and LCI, linked color imaging).

At Zuyderland Medical Center Sittard-Geleen, the Netherlands, a dataset with Olympus (Tokyo, Japan) images was collected between September 2022 and August 2023. In this center, images were taken by six endoscopists. The dataset contained one image in HDWL and one image in narrow-band imaging (NBI) for each colorectal polyp.

**Figure 1:**
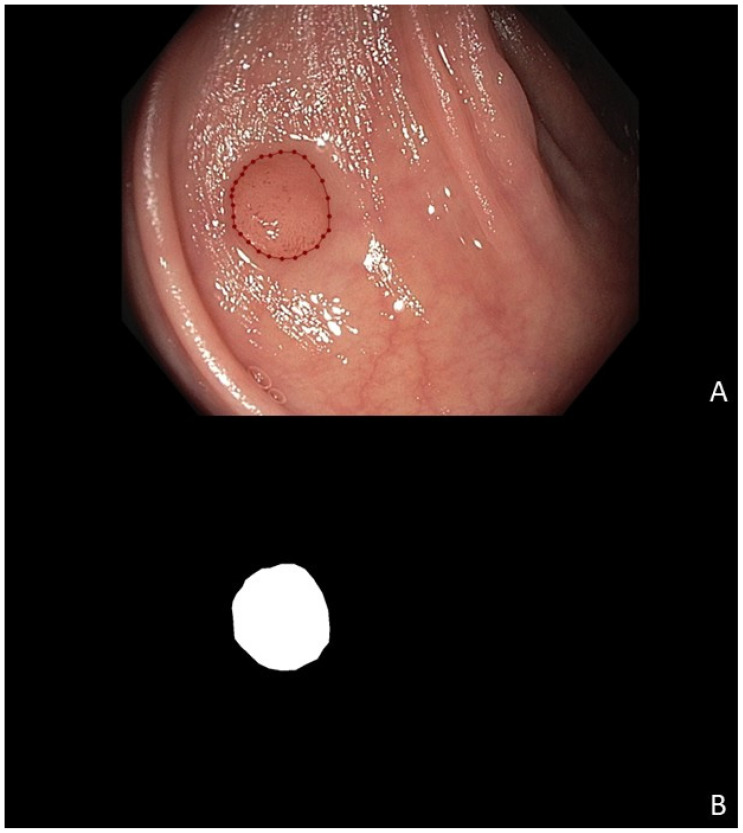
Example of human-annotated colorectal polyp position, showing (**A**) a labeled polyp and (**B**) the corresponding mask.

All polyps were marked in each image by a research physician (AT) using the annotation software LabelMe (Figure 1A) [15]. Subsequently, polyp masks were extracted from the annotated images (Figure 1B) to be used as a gold standard for polyp position. The Department of Electrical Engineering at Eindhoven University of Technology was responsible for the application of different network architectures to the data.

This study was approved by the Institutional Review Boardsof Bernhoven Hospital, MUMC+, and Zuyderland Medical Center and by the Medical Research Ethics Committee of MUMC+ (METC2019-1231 and METC2021-3036).

### 2.2. Network Architectures

Four AI algorithms were used in this study, with ResNet50 and EfficientNet-B4 serving as the core architectures for each algorithm. These models are known for achieving state-of-the-art accuracy on the ImageNet dataset. Algorithm 1 enhances the AI model employed in Dehghani et al. (2024) [16] by incorporating an in-domain pretrained model with a large-scale endoscopic dataset, namely GastroNet [17], while the other three algorithms (described in Kusters et al. (2022) [18]) rely on ImageNet pretraining but are trained with different amounts of images. Using these different AI algorithms enables an evaluation of how heatmap interpretability may be influenced by different model architectures (Algorithm 1 compared to the other algorithms) and training conditions, specifically varying dataset sizes (comparing Algorithms 2, 3, and 4). Various data augmentation techniques were applied during training to enhance the models’ generalization capabilities. A summary of the base architecture and the corresponding number of training images for each algorithm is presented in Table 1.

**Figure 2:**
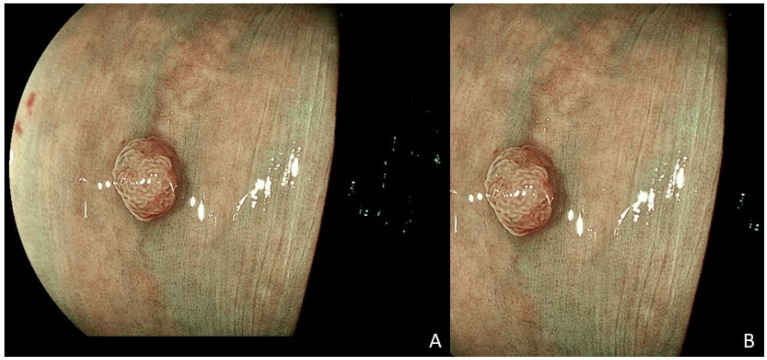
Example of (**A**) a training image and (**B**) the selected region of interest (ROI) from the central area of this image.

To optimize classification, the central region of the training images was automatically selected as the region of interest (ROI). This cropped area captures the polyp along with its surrounding texture, ensuring comprehensive coverage, as illustrated in Figure 2.

Each algorithm produced binary predictions (benign or premalignant), after which a heatmap was additionally extracted. Provided heatmaps are obtained using Grad-CAM [10]. Grad-CAM is a visualization technique that identifies parts of the image that mostly influenced the AI algorithm’s classification, with the most influential areas highlighted in red. This visual representation provides insight into how specific parts of the image impact the model’s output.

### 2.3. Statistical Analysis

Patient and polyp characteristics were described using mean and standard deviation (SD) values for numerical variables and number and percentage values for categorical ones.

Sensitivity, specificity, negative predictive value (NPV), positive predictive value (PPV), diagnostic accuracy, and area under the receiver operating characteristic curve (AUROC) with corresponding 95% confidence intervals [CIs] were calculated for all four algorithms, with histopathology as the gold standard. These 95% CIs were computed using cluster bootstrapping based on 5000 iterations to account for clustering (multiple images of the same polyp). For the analysis, polyp histology was dichotomized into the categories benign and premalignant. The category benign consisted solely of hyperplastic polyps. The category premalignant consisted of adenomas and sessile serrated lesions.

**Figure 3:**
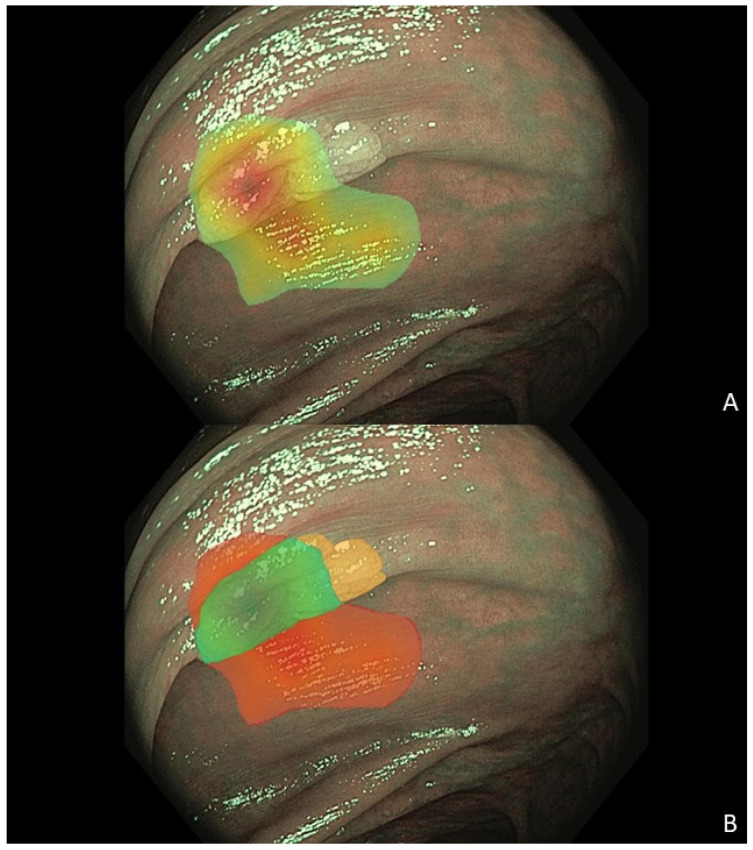
Example of a colorectal polyp image with a heatmap, showing (**A**) the heatmap on the polyp, (**B**) the part of the heatmap covering polyp in green, the part of the heatmap covering tissue surrounding the polyp in red, and the part of the polyp not covered by the heatmap in orange (3B).

The percentage of heatmap covering the polyp was calculated as the ratio between the overlap of the heatmap and the polyp (green in Figure 3B) and the joint heatmap area (red + green in Figure 3B), multiplied by 100.

The percentage of the polyp not covered by the heatmap was calculated as the ratio between the part of the polyp not covered by the heatmap (orange in Figure 3B) and the entire polyp area (green + orange in Figure 3B), multiplied by 100.

The relation between these two percentages and a correct algorithm prediction was evaluated using generalized estimating equations (GEEs) with a logit link. A GEE was used to account for the clustering of several images from the same polyp. Odds ratios (ORs), 95% CIs. corresponding original *p* values, and the adjusted *p* values using the Benjamini–Hochberg method are reported. Two-sided *p* values ≤ 0.05 were considered statistically significant. Statistical analyses were performed using IBM SPSS Statistics for Windows version 28 (IBM Corp., Armonk, NY, USA), R version 4.3.3, and the online Vassarstats calculator (https://vassarstats.net/kappa.html, accessed on 21 May 2024).

## 3. Results

### 3.1. Patients and Colorectal Polyps

In total, data from 195 patients were collected (Table A1). In most cases, one (49.7%) polyp per patient was collected.

From these 195 patients, 376 colorectal polyps were included in this study (Table 2). The majority of the colorectal polyps were diminutive (90.6%), with a mean size of 3.74 mm. Most polyps were tubular adenomas (79.5%). Benign, hyperplastic polyps represented 7.4% of the cases. Out of the 367 polyps, 212 (56.4%) were visualized with Fujifilm and the remaining 164 (43.6%) polyps with Olympus. On average, 5.7 images were available per polyp.

### 3.2. Diagnostic Performance of the Artificial Intelligence Algorithms

Out of the 2153 available colorectal polyp images, 20 images were excluded because the polyp was not visible in the cropped image version. The remaining 2133 images were diagnosed by four different AI algorithms (Table 3). Algorithm 1 showed the best diagnostic performance with a sensitivity of 80.6% (95% CI 77.1–84.0), specificity of 58.1% (95% CI 41.7–74.5), PPV of 97.0% (95% CI 95.4–98.7), NPV of 15.0% (95% CI 7.3–22.8), diagnostic accuracy of 79.3% (95% CI 76.0–82.7), and AUROC of 69.5% (95% CI 64.1–74.8).

### 3.3. Factors Associated with a Correct Algorithm Diagnosis

The results from GEE analysis examining the association between heatmap position and a correct algorithm diagnosis are presented in Table 4.

Higher percentages of heatmap covering the colorectal polyp were associated with a correct diagnosis in Algorithm 1 (OR 1.013 [95% CI 1.006–1.019], *p* < 0.001), Algorithm 2 (OR 1.025 [95% CI 1.011–1.039], *p* < 0.001), Algorithm 3 (OR 1.038 [95% CI 1.024–1.053], *p* < 0.001), and Algorithm 4 (OR 1.039 [95% CI 1.020–1.058], *p* < 0.001).

A higher percentage of the polyp not covered by the heatmap was associated with a correct diagnosis in Algorithm 1 (OR 1.006 [95% CI 1.003–1.010], *p* < 0.001). However, in Algorithm 2, a lower percentage of the polyp not covered by the heatmap was associated with a correct diagnosis (OR 0.992 [95% CI 0.985–1.000], *p* 0.044). Similar to Algorithm 2, Algorithms 3 and 4 showed negative, but not statistically significant, associations.

Polyps with a premalignant histopathology were associated with a correct algorithm diagnosis, with statistically significant results in three out of four algorithms.

Images from the endoscopy brand Olympus were also associated with a correct algorithm diagnosis, which was statistically significant in Algorithm 1 (OR 1.812 [95% CI 1.181–2.779], *p* 0.006) and Algorithm 4 (OR 1.481 [95% CI 1.006–2.180], *p* 0.046).

## 4. Discussion

In this study, we examined the association between heatmap position and the accuracy of four different algorithms in the characterization of colorectal polyps. This was examined both from the perspective of the heatmap and polyp, looking at the percentage of the heatmap covering colorectal polyp and looking at the percentage of the polyp that was not covered by heatmap.

GEE analysis showed a statistically significant association between a higher percentage of the heatmap covering the polyp and a correct diagnosis in all four AI algorithms. These findings indicate that it is clinically relevant to strive for AI algorithm predictions with a heatmap that covers as much colorectal polyp tissue as possible and as little surrounding colon tissue as possible. Therefore, it seems important for the CADx system to first correctly detect a colorectal polyp. Combining computer-aided detection (CADe) techniques with CADx systems could be preferred in future AI algorithms, similar to existing CADe/CADx combinations such as GI Genius (Medtronic, Dublin, Ireland) [19] and CAD EYE (Fujifilm, Tokyo, Japan) [20].

**Figure 4:**
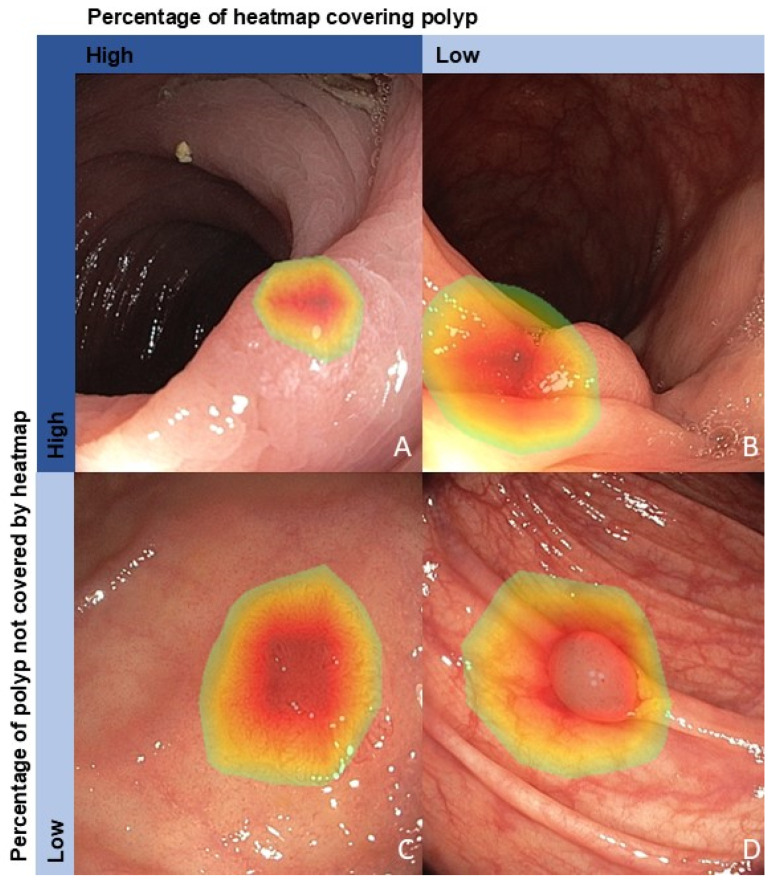
Examples of different heatmap coverage: (**A**) a heatmap covering only colorectal polyp tissue but missing part of the polyp, (**B**) a heatmap covering little colorectal polyp and much tissue surrounding the colorectal polyp, (**C**) a heatmap covering only colorectal polyp and little tissue surrounding the colorectal polyp, and (**D**) a heatmap covering the colorectal polyp tissue and covering much tissue surrounding the colorectal polyp.

**Figure A1:**
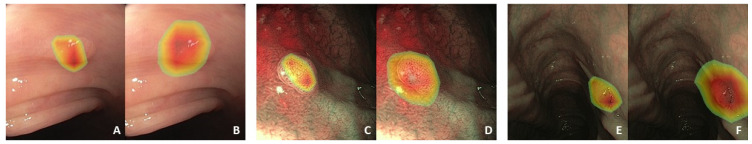
Examples of heatmap coverage from (**A**,**C**,**E**) Algorithm 1 and (**B**,**D**,**F**) Algorithm 2 in correctly classified cases.

In contrast, the percentage of a polyp not covered by the heatmap does not seem to be strongly associated with a correct diagnosis by the AI algorithms. In Algorithms 2–4, the expected association of a lower percentage of a polyp not covered by the heatmap and correct algorithm diagnosis was found, although only statistically significant in Algorithm 2 before using the Benjamini–Hochberg method. Unexpectedly, Algorithm 1 showed a statistically significant association between a higher percentage of a polyp not covered by the heatmap and a correct algorithm diagnosis. A visual comparison between Algorithms 1 and 2 demonstrated that Algorithm 1’s heatmaps appear more spatially focused, highlighting a smaller region within the lesion, whereas Algorithm 2 shows a broader coverage encompassing the entire lesion (Appendix A, Figure A1). It is important to consider that a higher percentage of a polyp not covered by the heatmap does not imply that Algorithm 1 mislocalizes the polyp, but rather that it uses a more refined focus on diagnostically relevant features. The different heatmap localization pattern of Algorithm 1 in comparison to the other algorithms can be a result of the different architecture and additional domain-specific self-supervised pretraining with GastroNet images (Table 1). This confirms the effectiveness of self-supervised learning to enhance AI algorithm robustness and interpretability, which has been shown in previous research in medical imaging [21]. The contradictory results for the different algorithms imply that, in general, the percentage of a polyp that is covered by the heatmap is not the most important indicator for a correct algorithm diagnosis, while the percentage of the heatmap that contains colorectal polyp tissue is. In Figure 4, examples of different heatmap coverages are visualized. In clinical practice, our results indicate that it seems the most important to aim for a heatmap that covers colorectal polyps (Figure 4A,C). It is less important if there is an additional part of the polyp not covered by that heatmap (Figure 4A). In case the heatmap covers more tissue surrounding the colorectal polyp (Figure 4B,D), the endoscopist using the AI algorithm in clinical practice could consider repositioning the endoscope and obtaining a new image as input for the AI algorithm, to possibly increase the chances of a correct algorithm diagnosis.

Additionally, our analysis showed a statistically significant association between polyp histology and a correct diagnosis in three out of four AI algorithms. This can be explained by the diagnostic performance of the algorithms. A relatively high sensitivity, when compared to a lower specificity, means that premalignant polyps are diagnosed correctly more often than benign polyps. This can be a consequence of a lower number of benign cases. We also found images with the endoscopy brand Olympus to be associated with correct diagnoses, indicating that the training data might be unbalanced, or this might possibly be as a consequence of pretraining with GastroNet in Algorithm 1 [17,22,23]. Other factors, such as age, gender, polyp location, and polyp size, did not appear to be associated with the chance of a correct diagnosis.

The application of AI in gastrointestinal endoscopy is increasing tremendously. However, endoscopists mostly lack the technical background to fully understand AI outcomes [22]. Ideally, the results of this study should be incorporated into a quality check with each AI algorithm diagnosis. Previous studies have shown the importance of image quality in relation to AI algorithm performance [24]. Factors such as blur and insufficient lighting can influence the performance of AI algorithms. Therefore, future algorithms would benefit from an image quality indicator with each prediction. If the heatmap does not sufficiently cover the polyp, this image quality indicator could advise the endoscopist to reposition the endoscope.

Visual explanations of AI algorithm predictions can be described as highlighting ‘important’ pixels, meaning that changes in the intensity of these pixels would impact the algorithm prediction the most [10]. Grad-CAM is a class-discriminative visualization technique, meaning that it localizes the prediction category within the image. This can also be modified into counterfactual explanations, highlighting regions that would cause the AI algorithm to change its prediction. In clinical practice, these counterfactual explanations could, for example, be used to point out feces or bubbles in the frame, which, after removal, would make the AI algorithm more confident in its classification. It should be noted that counterfactual explanations highlight all regions causing AI to change its predictions, which also include regions that are not always clinically logical. Noise can be a complicating factor in colorectal polyp images, caused by movements of the colon while capturing images of polyps, for example. Medical image denoising is an image preprocessing technique that has been shown to efficiently denoise radiological medical images and could potentially be applied to endoscopic images as well [25]. Deblurring has shown improved performance of AI models for several medical image analysis tasks [26]. Whether factors such as blur or inadequate lighting are also associated with less accurate heatmap positions is still unknown. The influence of denoising and deblurring on the accuracy of heatmaps should be considered in future research.

Even though heatmaps covering colorectal polyps are associated with correct algorithm diagnoses and can thus be used with this knowledge in clinical practice, we would like to emphasize the importance of the proper external validation of each AI algorithm [14]. Trust in the prediction of a CADx system should not merely rely on an accurate heatmap. Primarily, AI algorithm performance needs to be evaluated thoroughly. Subsequently, AI algorithms can be used in clinical practice with heatmaps as an indicator of algorithm accuracy, possibly increasing trust in the algorithm.

The limitations of this study should be addressed. Although the primary aim of this study was to investigate the association between heatmap position and AI diagnostic accuracy, the four algorithms used in this study showed suboptimal diagnostic performance. Even in suboptimal-performing models, understanding how and where the AI “looks” can provide meaningful insights into algorithm behavior and trustworthiness. It might be preferred to include more benign colorectal polyps to be able to assess specificity and NPV more precisely. However, it is important to note that the test dataset was collected prospectively and reflects clinical practice where hyperplastic polyps are less common and less frequently resected. This study aimed to preserve the clinical relevance of the results by avoiding the augmentation of the class imbalance in the test dataset. Other methods to improve algorithm performance could be to implement preprocessing techniques, to use large-scale pretraining and extend this to other algorithm architectures that have shown promise in medical imaging, such as Vision Transformers, or to use video data instead of static images.

Additionally, this study used independent datasets for AI algorithm training, internal validation, and final testing but lacks external validation. To further establish the robustness and generalizability of the results, external validation is an important direction for future research. With the limited number of correct diagnoses and eight variables in the GEE model, overfitting cannot be ruled out. Future research with external validation could clarify this.

Furthermore, 20 images had to be excluded from this study because the colorectal polyp was not visible in the cropped image. In future research, we hope to be able to apply the techniques on uncropped, original endoscopy images.

In this study, we used four different AI algorithms, aiming to obtain results that can be translated to many AI algorithms. However, we acknowledge that the association between heatmap position and diagnostic accuracy found in this study may still not be generalizable to other, better-performing algorithms. Before using an AI algorithm in clinical practice, it could be relevant to study this association for that particular algorithm.

Finally, multiple images from one polyp were used in the analysis. Corrections for this factor were applied in the calculations of the algorithm’s diagnostic performance. However, it could be preferred to use only one image per polyp, although creating large datasets with a single original image from one polyp can be challenging.

## 5. Conclusions

In conclusion, this study indicates that a higher percentage of heatmap covering polyp tissue is associated with a correct diagnosis of four different AI algorithms. Heatmap position was compared to the human-annotated polyp position. With these results, we hope to contribute to the optimal use of AI algorithms for colorectal polyps. Knowing how to interpret heatmaps has the potential to increase trust in AI and, with that, benefit the implementation of AI algorithms in clinical practice.

## Figures and Tables

**Table 1:** A summary of the base network architecture and the corresponding number of training images for each algorithm.

	Base Architecture	Number of Polyps	Pretraining
Algorithm 1	ResNet50	1359	ImageNet-GastroNet
Algorithm 2	EfficientNet-B4	1189	ImageNet
Algorithm 3	EfficientNet-B4	993	ImageNet
Algorithm 4	EfficientNet-B4	734	ImageNet

**Table 2:** Colorectal polyp characteristics.

Colorectal Polyp Characteristics	N = 376
Size in mm, mean (SD)	3.74 (3.1)
Size categories, n (%) ^1^	
Diminutive (≤5 mm)	337 (90.6)
Small (5–10 mm)	25 (6.7)
Large (>10 mm)	10 (2.7)
Location, n (%) ^2^	
Rectum	30 (8.0)
Sigmoid	89 (23.7)
Descending colon	37 (9.9)
Splenic flexure	2 (0.5)
Transverse colon	94 (25.1)
Hepatic flexure	11 (2.9)
Ascending colon	76 (20.3)
Cecum	36 (9.6)
Histology, n (%)	
Premalignant	
Tubular adenoma	299 (79.5)
Sessile serrated lesion	32 (8.5)
Tubulovillous adenoma	14 (3.7)
Traditionally serrated adenoma	3 (0.8)
Benign	
Hyperplastic polyp	28 (7.4)
Endoscopy brand, n (%)	
Fujifilm	212 (56.4)
Olympus	164 (43.6)
Images per polyp, mean (SD)	5.7 (3.4)

^1^ Polyp size was missing in four cases. ^2^ Polyp location was missing in one case. n, number; SD, standard deviation.

**Table 3:** Image-based diagnostic performance in predicting benign or premalignant conditions of 2133 colorectal polyp images with bootstrapping to account for clustering (multiple images of the same polyp).

	Algorithm 1	Algorithm 2	Algorithm 3	Algorithm 4
Sensitivity, % (95% CI)	80.6 (77.1–84.0)	75.3 (72.0–78.6)	65.3 (61.6–69.1)	79.4 (76.2–82.6)
Specificity, % (95% CI)	58.1 (41.7–74.5)	54.6 (39.2–70.1)	56.8 (39.2–74.5)	40.4 (27.8–52.9)
PPV, % (95% CI)	97.0 (95.4–98.7)	96.6 (94.5–98.6)	96.2 (93.8–98.6)	95.7 (93.5–98.0)
NPV, % (95% CI)	15.0 (7.3–22.8)	11.4 (6.0–16.9)	8.8 (4.5–13.1)	10.3 (5.5–15.1)
Diagnostic accuracy, % (95% CI)	79.3 (76.0–82.7)	74.1 (70.9–77.4)	64.8 (61.2–68.5)	77.2 (74.0–80.5)
AUROC, % (95% CI)	69.5 (64.1–74.8)	64.7 (59.3–70.1)	61.0 (55.7–66.3)	59.7 (54.1–65.3)

AUROC, area under the receiver operating characteristic curve; CI, confidence interval; NPV, negative predictive value; PPV, positive predictive value.

**Table 4:** Results of multivariable generalized estimating equation (GEE) analysis of factors associated with a correct diagnosis of four artificial intelligence algorithms trained to characterize colorectal polyps as benign or premalignant.

	Algorithm 1			Algorithm 2			Algorithm 3			Algorithm 4		
	OR [95% CI]	*p* Value	Adjusted *p* Value ^#^	OR [95% CI]	*p* Value	Adjusted *p* Value ^#^	OR [95% CI]	*p* Value	Adjusted *p* Value ^#^	OR [95% CI]	*p* Value	Adjusted *p* Value ^#^
Percentage of heatmap covering polyp	1.013 [1.006–1.019]	<0.001 *	0.003 *	1.025 [1.011–1.039]	<0.001 *	0.008 *	1.038 [1.024–1.053]	<0.001 *	0.008 *	1.039 [1.020–1.058]	<0.001 *	0.004 *
Percentage of polyp not covered by heatmap	1.006 [1.003–1.010]	<0.001 *	0.003 *	0.992 [0.985–1.000]	0.044 *	0.117	0.995 [0.989–1.001]	0.098	0.392	0.995 [0.986–1.004]	0.280	0.560
Endoscopy brand Olympus	1.812 [1.181–2.779]	0.006 *	0.012 *	1.328 [0.938–1.881]	0.109	0.218	1.220 [0.877–1.698]	0.237	0.539	1.481 [1.006–2.180]	0.046 *	0.123
Histology premalignant	4.002 [2.075–7.720]	<0.001 *	0.003 *	2.720 [1.465–5.049]	0.002 *	0.008 *	1.272 [0.605–2.674]	0.526	0.701	5.562 [2.909–10.636]	<0.001 *	0.004 *
Age	0.998 [0.971–1.025]	0.871	0.871	1.000 [0.979–1.022]	0.988	0.988	0.990 [0.971–1.010]	0.337	0.539	0.996 [0.971–1.021]	0.741	0.827
Female gender	1.071 [0.706–1.624]	0.746	0.853	0.859 [0.605–1.218]	0.392	0.523	1.022 [0.720–1.450]	0.903	0.903	1.045 [0.702–1.558]	0.827	0.827
Location polyp right-sided	1.282 [0.850–1.934]	0.237	0.379	0.857 [0.602–1.220]	0.392	0.523	0.834 [0.601–1.157]	0.276	0.539	0.945 [0.641–1.392]	0.774	0.827
Polyp size	0.980 [0.924–1.038]	0.490	0.653	1.007 [0.950–1.068]	0.807	0.922	1.014 [0.958–1.073]	0.633	0.723	1.019 [0.950–1.093]	0.599	0.827

^#^ Using the Benjamini–Hochberg method. * Significant *p* value < 0.05. OR, odds ratio; CI, confidence interval.

## Data Availability

The data are not publicly available due to contractual agreements regarding patient privacy with the hospitals where the data were collected.

## Abbreviations

The following abbreviations are used in this manuscript:

AI	Artificial intelligence
BLI	Blue light imaging
CADe	Computer-aided detection
CADx	Computer-aided diagnosis
GEE	Generalized estimating equations
Grad-CAM	Gradient-weighted class activation mapping
CI	Confidence interval
HDWL	High-definition white light
LCI	Linked color imaging
NBI	Narrow-band imaging
NPV	Negative predictive value
OR	Odds ratio
PPV	Positive predictive value

## Appendix A

**Table A1:** Patient characteristics.

Patient Characteristics	N = 195
Gender, n (%)	
Male	130 (66.7)
Female	66 (33.3)
Age, mean (SD)	65.8 (7.1)
Number of polyps per patient, n (%)	
1	97 (49.7)
2	47 (24.1)
3	28 (14.4)
4	15 (7.7)
5	7 (3.6)
6	1 (0.5)

n, number; SD, standard deviation.
